# The effects of active and passive smoking on selected trace element levels in human milk

**DOI:** 10.1038/s41598-023-48012-9

**Published:** 2023-11-25

**Authors:** Borhan Mansouri, Nammam Ali Azadi, Kiomars Sharafi, Samaneh Nakhaee

**Affiliations:** 1 Substance Abuse Prevention Research Center, Research Institute for Health, Kermanshah University of Medical Sciences, Kermanshah, Iran; 2 Biostatistics Department, School of Public Health, Iran University of Medical Sciences, Tehran, Iran; 3 Research Center for Environmental Determinants of Health (RCEDH), Research Institute for Health, Kermanshah University of Medical Sciences, Kermanshah, Iran; 4 Medical Toxicology and Drug Abuse Research Center (MTDRC), Birjand University of Medical Sciences, Birjand, Iran

**Keywords:** Health care, Medical research, Risk factors

## Abstract

Our study aimed to compare levels of six micro-elements and six potentially toxic elements in the breast milk of non-smoking women compared to those found in women who smoke tobacco and women exposed to second-hand smoke during pregnancy and lactation. This was a cross-sectional study conducted on 100 lactating women in western Iran. The studied subjects were in three groups: passive smokers, active smokers, and a control group. Concentrations of selected trace elements in breast milk (essential and non-essential metals) were determined using ICP-MS. Our results indicated that the parameters of education, fruit consumption, and cosmetics usage had a significant difference among the groups (*p* < 0.05). Moreover, for trace elements, the Kruskal–Wallis test was statistically significant for arsenic (As), cadmium (Cd), mercury (Hg), and lead (Pb) (*p* < 0.05). The post hoc Dunn test revealed a significant difference in the levels of As, Cd, Hg, and Pb between non-smoker and passive/active smoker groups (*p* < 0.05). Our findings illustrate that exposure to cigarette smoke can cause an increase in the level of potentially toxic elements in human milk, which is dangerous for the consumption of premature newborns, but more research is needed to evaluate the potential toxic mechanisms of toxic metals.

## Introduction

Breast milk is usually the only source of nutrition for infants during the first 6 months of life^1,2^. Human milk protects against a wide range of diseases and complications, such as respiratory illnesses, allergies, autoimmune diseases, and infant mortality. It also positively affects the development of intestinal microflora and reduces the severity of various infectious diseases^3,4^. Previous studies have shown that breastfeeding during infancy leads to reduced rates of infections and iron deficiency, lower obesity rates, and better intellectual performance in children^5,6^. However, human milk has been found to contain heavy metals, which pose a risk of toxic element accumulation and potential health effects for both infants and mothers^2,7^. Several studies have examined the composition of trace elements, such as iron, zinc, copper, and manganese in breast milk^8,9^, as excessive levels of these elements can have adverse health effects on breastfeeding infants^2^. While lead, cadmium, and arsenic have been extensively studied in human milk, high levels of manganese, cobalt, mercury, and chromium have also been reported^2^. Human breast milk can serve as a biological indicator of exposure to pollutants and is an important route for maternal excretion of trace elements^2^. Previous reports have highlighted the harmful effects of smoking and second-hand smoke (SHS) exposure on the composition of breast milk, which can reduce its protective properties and negatively impact infant health and development^3,10,11^. Tobacco smoke contains over 5300 compounds and 70 carcinogens. Breast milk from smoking mothers can expose infants to environmental compounds present in tobacco smoke^10^. Breastfed infants of smoking mothers are more likely to experience allergies, sleep disorders, increased colic, upper respiratory tract infections, cardiac rhythm disorders, and sudden infant death syndrome^3^. However, the exact underlying mechanisms behind these increased risks associated with smoke exposure are still being investigated^3^. Whileit was initially believed that the risks of smoking were limited to smokers themselves, it was determined in the 1980s that second-hand smoke is harmful to health even for non-smokers^11^, and metal concentrations differ between mainstream smoke inhaled by a smoker and sidestream smoke^3^. In the literature, some studies have assessed the concentration of different trace elements in the milk of lactating mothers worldwide^1,5,6,8,9,12–16^. However, studies specifically examining the effects of active and passive exposure to cigarette smoke on milk element concentration are limited^2,3,17^. Therefore, this study aimed to compare the levels of 6 micro-elements and 6 potentially toxic elements in the breast milk of non-smoking women with those who smoke cigarettes and women exposed to second-hand smoke.

## Materials and methods

### Study area

This cross-sectional study was conducted on lactating women from multiple health centers in the city of Kermanshah in western Iran. Milk samples were collected from 100 lactating women living in urban areas of Kermanshah from September to December 2021. The project received ethical approval from Kermanshah University of Medical Sciences (IR.KUMS.REC. 1400.589), and written informed consent was obtained from all participants. The inclusion criteria for the study were healthy nursing mothers with a normal and uncomplicated pregnancy who were primiparous and breastfeeding only one child. Exclusion criteria included mothers who were multiparous, had chronic pre-pregnancy diseases (cardiac or autoimmune diseases), lived in local areas with known or suspected pollutant emissions, and had not resided in their current area for at least the previous 5 years^10^.

### Sample collection

Two trained nurses collected milk samples according to study protocols. Collection was accomplished in the morning, 2 h after the previous breast-feeding. After washing their hands and chest area, each participant manually expressed 5–10 ml of milk in the morning. The samples were labeled and stored in falcon BD sterile polyethylene tubes at − 20 °C until further analysis.

### Sample digestion

Milk samples stored in the refrigerator were placed at room temperature to be ready for digestion. Then, 1 ml of milk was placed in test tubes, and 4 ml of nitric acid (HNO_3_, 65%, Merck, Germany) was added. The samples were left to digest slowly overnight at room temperature. The next day, 2 ml of 35% hydrogen peroxide (H_2_O_2_) was added to the samples, which were then placed in a water bath (TW12, Julabo GmbH, Germany) for 6 h or until the solution was clear at 98 °C. Following digestion, all samples were diluted with deionized water (18.2 MΩ cm at 25 °C, Fistreem, WSC044, UK) to a final volume of 25 mL. Inductively coupled plasma mass spectrometry (ICP-MS, Agilent 7900, Santa Clara, CA, USA) was used to measure the concentrations of magnesium (Mg), manganese (Mn), iron (Fe), cobalt (Co), copper (Cu), zinc (Zn), arsenic (As), cadmium (Cd), chromium (Cr), mercury (Hg), nickel (Ni), and lead (Pb) in the milk samples. The recovery rates of Mg, Mn, Fe, Co, Cu, Zn, As, Cd, Cr, Hg, Ni, and Pb were 97%, 98%, 102%, 96%, 98%, 99%, 97%, 102%, 96%, 98%, 99%, and 99%, respectively.

### Data analysis

Descriptive summaries are reported as median and interquartile range (IQR) values for numerical quantities. Categorical variables were reported as frequencies, and the association between two nominal variables was assessed using the chi-squared test Fisher exact test as appropriate. The Shapiro–Wilk test was used to evaluate the normality distribustion of data. Concentration levels between studied groups were compared using Kruskal–Wallis test followed by the Dunn test. Data analysis was performed using R version 4.0.2 (2020-06-22) software.

### Ethical approval

This study was conducted by the World Medical Association Declaration of Helsinki. This study was approved by the Research and Ethics Committee of Kermanshah University of Medical Sciences (IR.KUMS.REC. 1400.589). The written informed consent was obtained from lactating women referring to the health center to enter the study.

## Results

In this study, 100 lactating women were included and classified into three groups: passive smokers, active smokers, and a control group (Table 1). Based on the results, there were significant differences among the groups in their age, job, education, fruit consumption, and use of cosmetics (*p* < 0.05). However, no significant differences were observed in the consumption of salt, milk, vegetables, fast food, potatoes, and oil (*p* > 0.05).

**Table 1:** Characteristic of participating mothers.

Variable	Passive smokers n = 19	Active smokers n = 24	Non-smokers n = 57	*P*-value
Age	28.95 ± 4.95	33.96 ± 5.98	27.98 ± 6.93	**0.001**
BMI	26.76 ± 3.82	27.49 ± 3.72	26.78 ± 3.90	0.725
Job				
Housekeeping	6	31	6	**0.007**
Self-employed	4	16	13	
Employeed	9	10	5	
Education				
College	12	6	14	**0.005**
Less than college	7	18	43	
Salt use				
Low	13	20	48	0.342
High	6	4	9	
Fruit consumption				
Low	13	11	20	**0.039**
High	6	13	37	
Milk				
Low	12	12	31	0.683
High	7	12	26	
Vegetable				
Low	12	13	35	0.792
High	7	11	22	
Cosmetic usage				
Low	8	12	41	**0.031**
High	11	12	16	
Fast food				
Seldom	11	17	39	0.630
Often	8	7	18	
Potatoes intake				
< 4	9	11	22	0.726
> 4	10	13	35	
Cereal’s intake				
< 4	11	15	31	0.794
> 4	8	9	26	
Oil kind, in a week				
Solid	3	5	10	0.785
Liquid	4	7	20	
Mixture	12	12	27	

Significant values are in [bold].

The mean (standard deviation) concentrations of essential metals are presented in Table 2. Zinc (Zn) had the highest mean concentration, while cobalt (Co) had the lowest. The order of total mean of essential metals in breast milk was: Zn > Fe > Cu > Mg > Mn > Co. The passive group had higher average concentrations of trace elements Mg and Mn compared to the active group. If the result of the Kruskal–Wallis test was significant, the Dunn test was performed to determine differences between groups.

**Table 2:** The median and quartiles of the essential and Non-essential elements (µg/l) in the breast milk of Iranian lactating mothers.

Elements	IARC Evaluation of carcinogens	Smoker		Control	Total	*P*-value*
		Passive	Active			
Essential elements						
Mg	–	17.9 (5.65–29.45)	17.0 (11.50–22.17)	17.2 (11.00–29.40)	17.15 (10.62– 28.82)	0.892
Mn	–	4.4 (1.95–5.75)	4.2 (3.57–6.05)	5.4 (3.10–7.10)	4.75 (3.27–6.8)	0.340
Fe	–	254.7 (205.0–361.05)	291.7 (213.6–291.7)	291.2 (218.4–388.9)	390.15 (282.35–390.15)	0.769
Co	2B	0.5 (0.3–0.6)	0.6 (0.5–0.6)	0.5 (0.4–0.6)	0.5 (0.4–0.6)	0.138
Cu	–	23.3 (14.60–30.50)	20.2 (7.95–26.67)	18.4 (9.80–29.40)	20.85 (8.92–28.37)	0.776
Zn	–	378.3 (97.90–653.35)	381.2 (281.2–921.8)	450.9 (225.4856.3)	427.5 (223.9–858.07)	0.443
Non–essential elements						
As	1	1.48 (1.09–2.69)	2.96 (1.19–4.34)	0.71 (0.12–1.80)	1.19 (0.36–3.10)	**< 0.001**
Cd	1	0.88 (0.76–1.14)	0.86 (0.68–1.16)	0.60 (0.26–0.77)	0.69 (0.37–1.1)	**< 0.001**
Cr	1	30.68 (24.06–44.74)	41.06 (31.85–45.46)	31.092 (20.58–53.30)	34.29 (22.44–48.43)	0.440
Hg	3	0.34 (0.24–0.59)	0.44 (0.27–0.44)	0.14 (0.11–0.14)	0.14 (0.14–0.45)	**< 0.001**
Ni	1	18.31 (13.32–22.97)	18.77 (15.09–22.25)	15.36 (11.30–22.42)	17.99 (11.87–22.46)	0.586
Pb	2B	14.09 (9.73–17.51)	14.24 (10.83–17.86)	8.99 (7.36–11.80)	11.01 (7.80–14.51)	**0.0004**

*Following the lack of normality distribution of the data, Kruskal–Wallis test was used to compare concentrations between groups. Results are given as median (Q1–Q3). Magnesium (Mg), Manganese (Mn), Iron (Fe), Cobalt (Co), Cupper (Cu), Zinc (Zn), Arsenic (As), Cadmium (Cd), Chromium (Cr), Mercury (Hg), Nickel (Ni), lead (Pb).

Significant values are in [bold].

**Figure 1:**
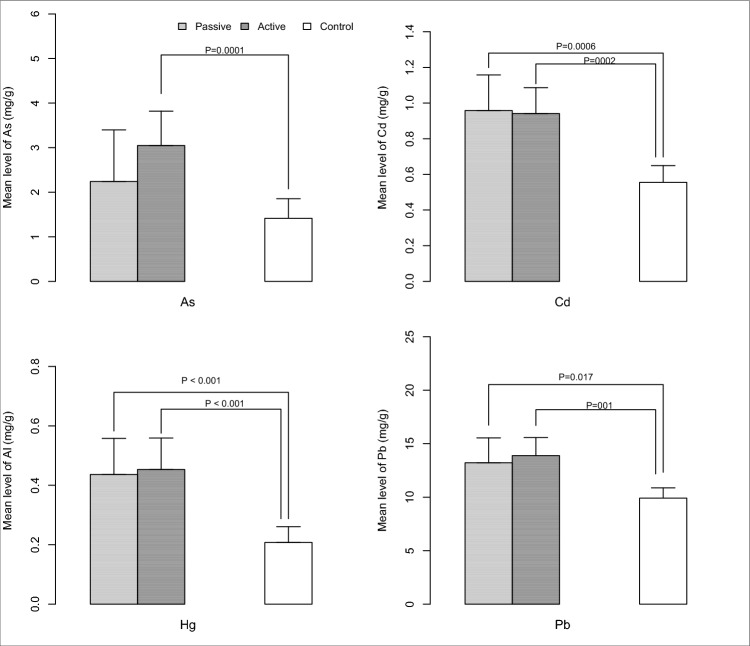
Post-hoc Dunn test controlled for Type I error revealed a significant difference in the levels of As, Cd, Hg, and Pb (µg/l) between non-smoker and passive/active smoker groups.

The mean (standard deviation) concentrations of non-essential metals are also presented in Table 2. Chromium (Cr) had the highest mean concentration, while mercury (Hg) had the lowest. The order of total mean of non-essential metals in breast milk was: Cr > Ni > Pb > As > Cd > Hg. According to the results in Table 2, the mean concentration of non-essential metals (except Cd) was higher in the active group compared to the passive group. For non-essential elements, the Kruskal–Wallis test was significant for As (Chi-square = 17.0, *p* = 0.0002, df = 2, effect size η^2^ = 0.172), Cd (Chi-square = 22.12, *p* < 0.001, df = 2, η^2^ = 0.223), Hg (Chi-square = 26.71, *p* < 0.001, df = 2, η^2^ = 0.27), and Pb (Chi-square = 15.31, *p* < 0.001, df = 2, η^2^ = 0.155). Post hoc Dunn tests adjusted for type I error revealed significant differences in As levels between non-smokers and active smokers (Z = − 4.06, *p* < 0.001), Cd levels between non-smokers and passive smokers (Z = − 3.51, *p* < 0.001) and non-smokers and active smokers (Z = − 3.97, *p* < 0.001), Hg levels between non-smokers and passive smokers (Z = − 4.04, *p* < 0.001) and non-smokers and active smokers (Z = − 4.20, *p* < 0.001), and Pb levels between non-smokers and active smokers (Z = − 3.56, *p* = 0.001) and non-smokers and passive smokers (Z = − 2.53, *p* = 0.017) (Fig. 1).

## Discussion

Tobacco smoke contains thousands of toxic and carcinogenic elements that people may be exposed to in different public places. Exposure to cigarette smoke, either directly or indirectly, has harmful effects on human health and increases the risk of various diseases^18^. In the present study, the effects of active and second-hand smoking on the milk concentration of different essential and toxic elements were investigated. We found that the levels of Cd, Pb, and Hg were significantly higher in the breast milk of passive and active smokers compared to nonsmokers also there was a significant difference in the levels of As between non-smoker and active smoker groups. Several trace elements are considered potentially toxic, and child exposure to these elements is related to anemia, cancers, interference with bone growth, adverse effects on the nervous system, etc.^2^. WHO/FAO has established provisional tolerable weekly intake (PTWIs) for different toxic metals, which are 7, 25, and 5 µg/kg for Cd, Pb, and total Hg, respectively^1^.

Smoking can contribute to increasing the level of toxic metals in breast milk^16^. Infants and children are particularly at risk of toxic metal accumulation as a result of lower body weight, slower excretion, as well as decreased immunity^16^. The heavy metal absorption in infants is usually greater when on a milk diet, possibly due to binding to easily absorbed proteins of milk^1^. The elevated levels of heavy metals in milk may interfere with the function of bioactive substances essential for the optimal development and growth of infants and children^10^. Killian et al.^19^ have reported that cadmium and lead increase oxidative stress and have an adverse synergistic effect on metabolic pathways in children.

One of the most important concerns is cadmium (classified as a Group1 carcinogen by the IARC), which is a toxic metal that disrupts the metabolism of some micronutrients such as iron, copper, zinc, and magnesium^10,20,21^. Maintaining proper levels of these microelements is critical for normal infant development^3^. Cadmium is a metallic element found naturally in the environment and as a pollutant. It can contaminate soil and be absorbed by crops, which can then be consumed by people through diet and smoking. However, recent data shows that Cd concentrations in crops and food are decreasing. Foods like cereals, vegetables, and shellfish are the main sources of Cd intake in humans. Rice is a significant source of Cd exposure in many countries. About 5% of Cd ingested in food is absorbed^22–24^. In some studies, a tendency to higher milk Cd levels was observed in smokers, suggesting that level of Cd in breast milk is influenced by the smoking behaviors of the mothers^12^. In this regard, one study showed that milk Cd concentration in smoking mothers during lactation increased with increasing cigarette consumption^17^. A study has revealed that Cd concentrations in “transitional milk” (milk created following colostrum production) were about four times higher in smoker mothers than in non-smokers^20^. Grzunov Letini´c et al.^14^ have documented the negative effects of smoking on Cd concentrations in the blood, mature milk, and transitional milk of women who smoke. For the effects of second-hand smoking in the literature, Cd, has been shown to elevate in the breast milk of women exposed during pregnancy to cigarette smoke^3^. In contrast, another study showed that Smoking habits in the family (mother's smoking during and/or before pregnancy, smoking of father) increased the milk Cd levels, but the differences were not statistically significant^1^.

Human exposure to lead has grown dramatically. It is counted to be one of the hazardous environmental exposures. Potential Source for lead exposure includes lead-based paints, occupational exposures, air, water, soil, dust, food (particularly milk, fish, flour, vegetables, tea, lemon juice, tomato paste, and rice), toys, dried herbs/herbal medicine, makeup products, old metal pipes, adulterated opium and smoking^25,26^. Based on the results of Ursinyova et al.^1^, smoking habits have no significant effect on breast milk lead content. The Pb levels in milk were slightly higher in mothers who smoked before pregnancy than in non-smokers (5.13 vs. 4.51 µg/kg), and the differences were not significant^1^. On the other hand, another study showed that smoking significantly increased milk Pb levels^13^. Also, Grzunov Letini´c et al.^14^ have recorded the negative effects of tobacco smoking on Pb concentrations in transitional milk. For the effects of second-hand smoking in the literature, Szukalska et al.^3^ confirmed that tobacco smoke exposure increases the Pb concentration of colostrum and mature milk. Pb levels in breast milk reflect both endogenous and exogenous exposure to this toxic metal. Published data have documented that pregnancy and breastfeeding increase bone turnover, and thus increase the movement of Pb from the mother's skeleton^2,27^.

Similarly to Pb, the fetus and children are more sensitive to Hg than adults. In humans the target of Hg toxicity is the nervous system and the kidneys, depending on its chemical form^1^. Some factors were observed to be responsible for mercury pollution, including food (mainly fish and canned food, although rice may be another source of methylmercury for Asians), amalgam dental fillings, contact lenses, smoking, cosmetics, medical instruments, chlor-alkali industries, steel industries, cement plants, plastic industries, certain agriculture, and pharmaceutical industries^28–30^. Yalçin et al.^31^ report that passive/active smoking during pregnancy increased Hg concentrations in breast milk. Another study also revealed that smoking habits during pregnancy and the number of cigarettes smoked increased Hg levels significantly^32^. In contrast, Örün et al.^15^ Found that exposure to cigarette smoke during pregnancy and the second month after delivery did not affect the Hg concentrations of breast milk.

One of the limitations of this study is its small sample size, which may limit the generalizability of the findings. Additionally, the cross-sectional design only allows for an observation of associations and does not establish causality. Therefore, further research with larger sample sizes and longitudinal designs is needed to confirm and extend these findings. In future studies with a larger number of women, the evaluation of toxic metal concentrations depending on the amount of tobacco exposure is recommended. Public health information and messaging are essential to inform pregnant women about the adverse effects of cigarette smoking during pregnancy and especially during breastfeeding. This information should also include the possible adverse effects of maternal secondhand smoke on the composition of breast milk.

## Conclusion

In conclusion, the passive and active smoking of mothers significantly increased Cd, Pb, and Hg levels of human breastmilk. These findings support the necessity for increased concern and information for lactating women about avoiding passive or active exposure to cigarette smoke due to its negative effects on breast milk with additional risks to their infants.

## Data Availability

The datasets used and analyzed during the current research are available from the corresponding author upon request.
